# The effect of multi-level HIV prevention interventions on common mental disorders among adolescents and young adults in rural South Africa

**DOI:** 10.1371/journal.pgph.0005183

**Published:** 2025-12-11

**Authors:** Nondumiso Mthiyane, Guy Harling, Andrew Copas, Bianca De Stavola, Natsayi Chimbindi, Lorraine Sherr, Thembelihle Zuma, Jaco Dreyer, Xanthe Hunt, Nothando Ngwenya, Maryam Shahmanesh

**Affiliations:** 1 Africa Health Research Institute, KwaZulu-Natal, South Africa; 2 Institute for Golbal Health, University College London, London, United Kingdom; 3 MRC/Wits Rural Public Health & Health Transitions Research Unit (Agincourt), University of the Witwatersrand, South Africa; 4 University of KwaZulu-Natal, Durban, South Africa; 5 South African Medical Research Council, Cape Town, South Africa; 6 Stellenbosch University, Stellenbosch, South Africa; 7 Department of Psychiatry, University of Oxford, United Kingdom; PLOS: Public Library of Science, UNITED STATES OF AMERICA

## Abstract

This study evaluated the effect of multi-level HIV prevention interventions delivered through DREAMS (Determined, Resilient, Empowered, AIDS-free, Motivated, and Safe) program on common mental disorders (CMD) among adolescents and young adults (AYA) in rural South Africa. We used longitudinal data from two cohorts of AYA aged 13–35 years (N = 2184 for cohort 1 and N = 3067 for cohort 2), recruited between May 2017 and December 2018, with data collected at baseline and follow-up surveys until 2019. Retention rates exceeded 75% in both cohorts. Multi-level HIV prevention interventions included individual-level services like HIV testing and counselling, and post-violence care, and community-level interventions such as social asset building, social protection, and school-based HIV education. Probable CMD was measured using a 14-item Shona Symptoms Questionnaire, with a score of ≥9 indicating CMD. We used inverse probability of treatment weighting adjusting for socio-demographics, food insecurity, experienced violence, sexual behaviour, HIV status and baseline CMD, to estimate the average effect of multi-level interventions on CMD, quantified as a risk difference (RD). Of the 3944 participants, 519 received no intervention, 1060 received community-level interventions, 814 received individual-level interventions and 1551 received multi-level interventions. The prevalence of CMD increased with age, reaching 27% among those aged <25 years. In the causal analysis, exposure to multi-level interventions relative to no intervention was estimated to increase CMD (RD = 4.59, 95% confidence interval (CI):1.61-7.78). Additionally, when comparing multi-level interventions to community-level interventions alone, community-level interventions were found to be protective against CMD among adolescent girls and young women in Cohort 1 (RD = 5.45, 95% CI:1.54-8.92), indicating a higher CMD risk in the multi-level intervention group. These findings suggest that multi-level HIV prevention interventions as currently delivered, may not meet mental health needs in vulnerable AYA. Mental health screening and care should be integrated into HIV services for early diagnosis and treatment of CMD.

## Introduction

Common mental disorders are prevalent among young people in low and middle-income countries (LMICs) and are often untreated [1]. Mental disorders such as depression and anxiety contribute significantly to the global disease burden [2]. In Africa, young people are at a greater risk of CMD, partly because of their heightened risk for HIV [3–5]. They also face multiple challenges including poverty and different forms of violence which contribute to poor mental health and HIV risk [6,7]. In particular, financial hardships that lead to psychological distress have been shown to be linked to the extent to which young people engage in unsafe behaviours [8–10]. Exposure to violence including but not limited to gender-based violence and violence against children, are established risk factors for mental health disorders and increase vulnerability to HIV [11,12]. Given the bidirectional relationship between mental health and HIV, interventions that can address both these conditions should be considered.

Combination HIV prevention has been effective in reducing factors that increase vulnerability to HIV among youth [13–16]. Behavioural and structural interventions have been shown to reduce HIV risk by promoting safe sexual behaviours, economic empowerment, social support, women empowerment and gender equity [17,18]. In this setting, we have shown that exposure to both individual and community-level HIV prevention interventions was associated with subsequent condom use and voluntary medical male circumcision [13]. Despite the promising results, the evidence on whether this multi-level approach to HIV prevention may be useful in improving mental health in adolescents and young adults living in high HIV settings is lacking.

On the other hand multi-level interventions (delivered to groups and/or individuals) that incorporate economic empowerment, peer support and cognitive behavioural therapy have been shown to be effective in reducing common mental disorders among vulnerable adolescents in sub-Saharan Africa [19–23]. However, this evidence is limited to vulnerable young people who are already experiencing symptoms or are at high risk of CMD, highlighting the gap in preventive measures for mental health. In resource-limited settings, where mental health care may not be affordable or available, multi-level preventive interventions may be vital in reducing mental health disorders and the risk of HIV.

This study used the opportunity of multi-level HIV prevention interventions that were provided through DREAMS (Determined, Resilient, Empowered, AIDS-free, Motivated and Safe) partnership aimed at reducing HIV infections among adolescent girls and young women (AGYW), to explore the effect on mental health of adolescents and youth. DREAMS delivered packages of interventions to young people, their families and communities. The packages included (a) individual-level interventions aimed at promoting safe sexual behaviours and (b) community-level interventions (contextual) aimed at building resilience, problem-solving skills and self-esteem [24]. We hypothesised that young people who participated in multi-level interventions that combined individual-level intervention and community-level intervention would report low CMD symptoms relative to non-participants or those who received single-level interventions (either community-level interventions only or individual-level interventions only).

## Methods

### Study design and setting

We used data from two cohort studies that evaluated the impact of DREAMS among AGYW aged 13–22 (Cohort 1), and males aged 13–35 and females aged 24–29 (Cohort 2). These two cohort studies were conducted in the southern area of the Africa Health Research Institute Health and Demographic Surveillance System (HDSS) located in uMkhanyakude district of KwaZulu-Natal. The HDSS covers an area of 438 km^2^, with a population of approximately 100,000 people who are members of 12,000 households since 2000 [25]. The area is largely rural with one town with an approximate population of 30,000 people. AHRI conducts annual household-based surveys to collect information on births, deaths, and migration patterns among all household members. In addition, residents aged ≥15 years are invited to participate in an annual HIV serosurvey, and to complete a questionnaire on general health and sexual behaviour.

A sample of AGYW aged between 13–22 years (Cohort 1) was randomly selected from a list of age-eligible residents recorded in the HDSS in 2017. A random sample was stratified by age and by 45 geographic areas (blocks). Similarly, a random stratified sample of adolescent boys and young men (ABYM) aged 13–35 and young women (YW) aged 24–29 (Cohort 2) was selected in 2018. A sample was stratified by age, gender (male and female) and by 45 geographic areas. All sample size calculations accounted for the 30–40% of age-eligible residents recorded in the HDSS who were no longer eligible at the time of enrolment due to outmigration or death. Probability proportional to size sampling (PPS) without replacement was used to ensure that the selected samples represent different sizes of sampling units (age group and geographic area).

### Data collection and management

Between May 2017 and December 2018, we recruited eligible participants from their homes and followed them up until 2019. Data collection for Cohort 1 took place from 2017 to 2019, and for Cohort 2, from 2018 to 2019. The data were collected by a team of trained research assistants using face-to-face and self-administered interviews and captured electronically in RedCap software [26]. A structured questionnaire was used to collect data on general health, uptake of DREAMS interventions, behaviour and sexual relationships. The questions about sexual relationships were self-administered by a participant, and research assistant was available to support the participant if needed. The same sets of questions were asked at baseline and during follow-up surveys. Datasets from two cohort studies were appended to form one dataset and were merged (using household unique identifier and year of survey) with other household datasets containing migration history and household assets information. The data included a maximum of three data points from 2017 to 2019, with both exposure and outcome variables measured in each survey.

### Measures

#### Outcome measure.

The outcome of interest was probable common mental disorders (CMD). This was measured using the 14-item Shona Symptom Questionnaire which was developed and validated in Zimbabwe [27,28]. The tool asks about the symptoms experienced in the past seven days. The outcome was measured among participants who responded to all SSQ-14 items. All ‘yes responses’ were given a score of 1 and summed to create a final score for each individual. A binary variable indicating whether a participant scores above a validated cut-off (≥ 9) was created [28]. According to the validated cut off, this score indicated mild, moderate, and/or severe mental disorder. For the purpose of this analysis, we considered the outcome measured in 2019 (last follow-up).

#### Exposure measure.

There were nine community-level interventions and seven individual-level interventions that were supported by DREAMS in uMkhanyakude. Detailed information on the interventions is provided elsewhere [13,29]. Community-level interventions included social assets building interventions (safe spaces, mentor program, social assets program), community mobilisation and gender norms (violence prevention, school-based HIV education) and social protection (cash transfers and parenting program).

For the purpose of this analysis, the individual-level interventions tested in this analysis included only HIV testing and counselling services and post-violence care as described in Table 1. These two individual-level interventions were selected because they involve some degree of individual counselling which could hypothetically also support mental health. It should, however, be noted that HIV counselling that is relevant to mental health may only be provided to individuals who test positive for HIV or affected by HIV (through their sex partners, friends or family members). HIV testing and counselling services consists of different types of HIV testing (facility-based, home-based, self-testing, partner testing), linkage to antiretroviral therapy and support for ART adherence. Post-violence care services were delivered in collaboration with health facilities, police and social development department and included psychosocial support, HIV testing and counselling services (includes post exposure prophylaxis) and contraception. The psychosocial support involved the following:

**Table 1:** Description of interventions supported by DREAMS in uMkhanyakude used in this analysis.

Intervention	Description	Eligible population^a^
**Community-level**		
Financial literacy training^b^	Includes saving groups and microfinance program and ASPIRES	All
Vocational/business skills training^b^	Helps young people to get the essential skills needed to start a business.	All
Local program for parenting/caregiving^b^	Improves parent-child relationships and communication and reduce problem behaviours and emotional distress for both parent/caregiver and child (TALC)	All aged 13–19
Cash transfers	Support for school fees, government social grants or unconditional cash transfers to families.	All
Safe spaces^b^	Places where AGYW meet regularly to talk about their health and other challenges they face in their lives.	AGYW aged 13–24
Mentor program^b^	Offered in safe spaces where trained mentors meet with groups of girls to discuss economic hardships and health-related challenges.	AGYW aged 13–24
Social assets program^b^	Helps AGYW build strong relationships with their peer and adults who can offer emotional and material support. Vhutshilo 1&2	AGYW aged 13–24
HIV education or Life skills	Offered in all schools as part of basic education curriculum. Participants for this intervention included adolescents aged <20 years.	13-19 years
Gender norms and violence prevention related programs	Include gender-based violence prevention and gender norms-related education and sexual and reproductive health communication and relationship skills. Offered to adolescents in schools and to all age groups in communities (stepping stones).	All
**Individual-level**		
HIV testing and counselling services	Include all types of HIV testing (facility-based, mobile and home-based testing).	All
Post-violence care	Designed for victims of violence (sexual, physical, and emotional abuse).	All

^a^Participants eligible for intervention and included in the analysis, ^**b**^ Interventions which were only provided through DREAMS.

- Establishing victim’s psychological and physical safety concerns by referring them to other service providers,
- Providing long-term support to victims and their immediate families,
- Offering counselling and support to victims for court appearances, and
- Facilitating support group sessions to increase victims’ knowledge and awareness and to empower them.

The exposure to intervention was defined as participation in individual and community-level (group-based) interventions in the past 12 months. Using data collected in 2018 (ensuring that exposure was measured before the outcome), we created an exposure variable with four participation groups: no intervention; community-level only; individual-level only which included HIV testing and counselling and post-violence care; and multi-level (community and individual-level) interventions. Two binary exposure variables were created to compare multi-level interventions with community-level and individual-level interventions separately. Participants who did not receive any interventions were excluded in these binary variables. The first binary variable compared multi-level interventions with community-level interventions, while the second variable compared multi-level interventions with individual-level interventions.

#### Confounders.

**Fig 1:**
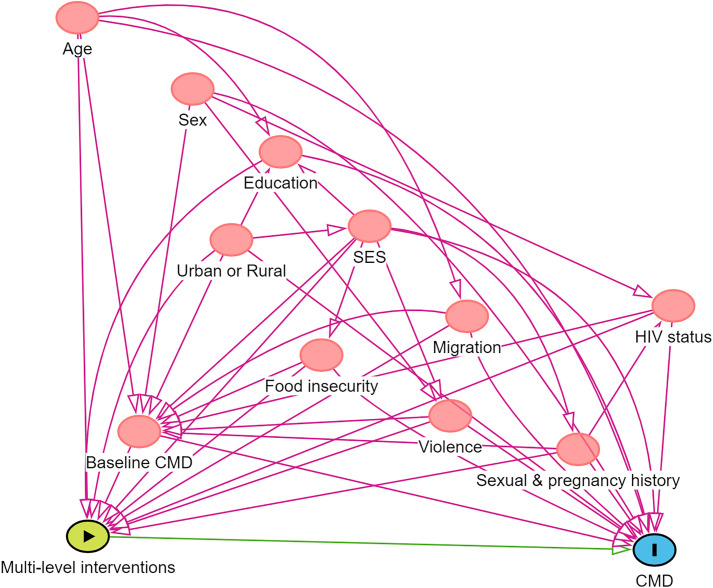
Directed Acyclic Graph describing causal relationships between multi-level interventions and CMD.

The set of confounders included in the analysis was informed by the literature and the available data on these variables. We used a directed acyclic graph (DAG) to describe the hypothesised relationships between exposure and outcome, and identified a minimal sufficient adjustment set of confounders for estimating the total effect of multi-level HIV prevention interventions on CMD at the last follow-up (Fig 1). Potential confounders included socio-demographic variables (age, gender, geographic area, household wealth index, education, household food insecurity, migration), sexual behaviour and HIV status. Geographic area was classified as either rural or urban (including townships) location. Household wealth index scores were calculated using principal component analysis (PCA) based on the ownership of household assets and access to safe drinking water and sanitisation. The scores from a first principal component were divided into tertiles.

For education, the highest level of education achieved was categorised as primary, secondary or completed secondary/tertiary. Food insecurity was defined as any report of reducing the size of food potions or skipping meals by any member of a household because there was not enough money to buy food in the past 12 months. Migration was defined as having moved home since age of 13. For pregnancy and sexual history, a composite variable was used, coded as 0 if the participant had never had sex, 1 if they had ever had sex but were never pregnant and 2 if they had ever been pregnant. We also included baseline CMD as a confounding factor. All confounding variables were measured at baseline (2017 for Cohort 1 and 2018 for Cohort 2).

### Statistical methods

We described baseline characteristics of young people using frequencies and percentages. Cronbach’s alpha coefficient was used to test for consistency and reliability of SSQ-14 items. We described the prevalence of CMD at baseline and endline by age and sex using percentages.

We estimated the average treatment effect (ATE) which is the average effect of the intervention in the entire target population using inverse probability of treatment weighting (IPTW). Prior to estimating average treatment effect (ATE), we fitted logistic regression models adjusting for potential confounders, to assess whether the exposure to multi-level interventions was associated with CMD. First, we fitted the exposure variable as categorical with four levels (no intervention, community-level only, individual-level only and multi-level intervention). Lastly, we fitted binary exposure variables (multi-level vs community-level only and multi-level vs individual-level only) in two separate models. The three types of analysis were repeated by cohort (Cohort 1 and Cohort 2).

The ATE was estimated in two steps. First, we fitted multinomial logistic regression models in which participation in multi-level HIV interventions, community alone, or individual alone, or none was included as an outcome, with covariates as explanatory variables, to estimate propensity scores. Covariates in the propensity score model included socio-demographics, food insecurity, experienced violence, sexual behaviour, baseline CMD and HIV status as outlined in the DAG. These variables were selected based on existing literature demonstrating their associations with both CMD and exposure to DREAMS interventions. Based on propensity scores, individuals were weighted by the inverse probability of receiving the intervention they received.

We evaluated the distribution of propensity scores using plots and implemented a trimming approach by excluding observations (n = 81, 2.1%) with propensity scores below the 1^st^ percentile and above the 99^th^ percentile [30] (see S1 Fig, S2 Fig and S3 Fig). Subsequently, stabilised weights were calculated. Balance and overlap of measured covariates were assessed as recommended by Rubin 2008 [31]. Covariate balance before and after weighting was assessed using standardised mean differences (SMDs), with visualizations presented as love plots (see S1 Table, S4 Fig, S5 Fig and S6 Fig). As some covariates remained imbalanced (SMD > 0.1) after weighting, all covariates were included in the outcome model to address potential residual confounding. Second, the propensity scores were used to weight individuals in each group by the inverse probability of receiving the intervention they received in a second binary logistic model, estimating the predicted probabilities of CMD for exposed and non-exposed individuals.

Lastly, the effect of interventions was measured as an absolute percent difference in predicted prevalence of CMD. This refers to the difference in average predicted probability of CMD between the scenarios where all participants received individual-level, community-level, or both interventions and the scenario where no participants received any intervention. Confidence intervals were computed by employing a bootstrap method, where the estimation process outlined earlier was repeated in 1000 samples drawn with replacement from the entire dataset. Same steps were repeated using binary exposure to estimate the ATE of multi-level interventions relative to the single-level interventions. The analysis was conducted in both cohorts combined and repeated separately by cohort (Cohort 1 and Cohort 2), to assess whether the estimated effects are influenced by study duration which differed by cohort. To assess the robustness of our estimates to potential unmeasured confounding, we conducted a sensitivity analysis by calculating the E-value [32]. The E-value quantifies the minimum strength of association that an unmeasured confounder would need to have with both the intervention and the outcome, beyond the measured covariates, to fully explain away the observed intervention effect. A higher E-value (greater than 2) suggests that the observed association is less likely to be explained by unmeasured confounding alone. We also used a propensity score-based regression adjustment method, adjusting for the propensity scores alongside the covariates.

We used a complete-case approach, as the amount of missing data was minimal, ranging from 0.1% to 0.3%, and was limited to only four variables out of 14 variables included in the analysis. For the household wealth index and sexual behaviour variables, missing data were coded as “unknown,” and no participants were excluded due to missingness in these variables. All analyses were performed using Stata version 18 (StataCorp LP, College Station, Texas USA) and R version 4.5.1 [33].

### Ethics

Ethical approvals were obtained from the University of KwaZulu-Natal Biomedical Research Ethics Committee (BFC339/19), the London School of Hygiene & Tropical Medicine Research Ethics Committee (REF11835) and the AHRI Somkhele Community Advisory Boards. Additional ethical approval for secondary data analysis was attained from University College London (18321/001). Potential participants were visited in their homes and invited to participate in the study. Participants aged 18 years or older provided written consent. Parental consent with participant written assent was sought for participants younger than 18 years.

## Results

**Fig 2:**
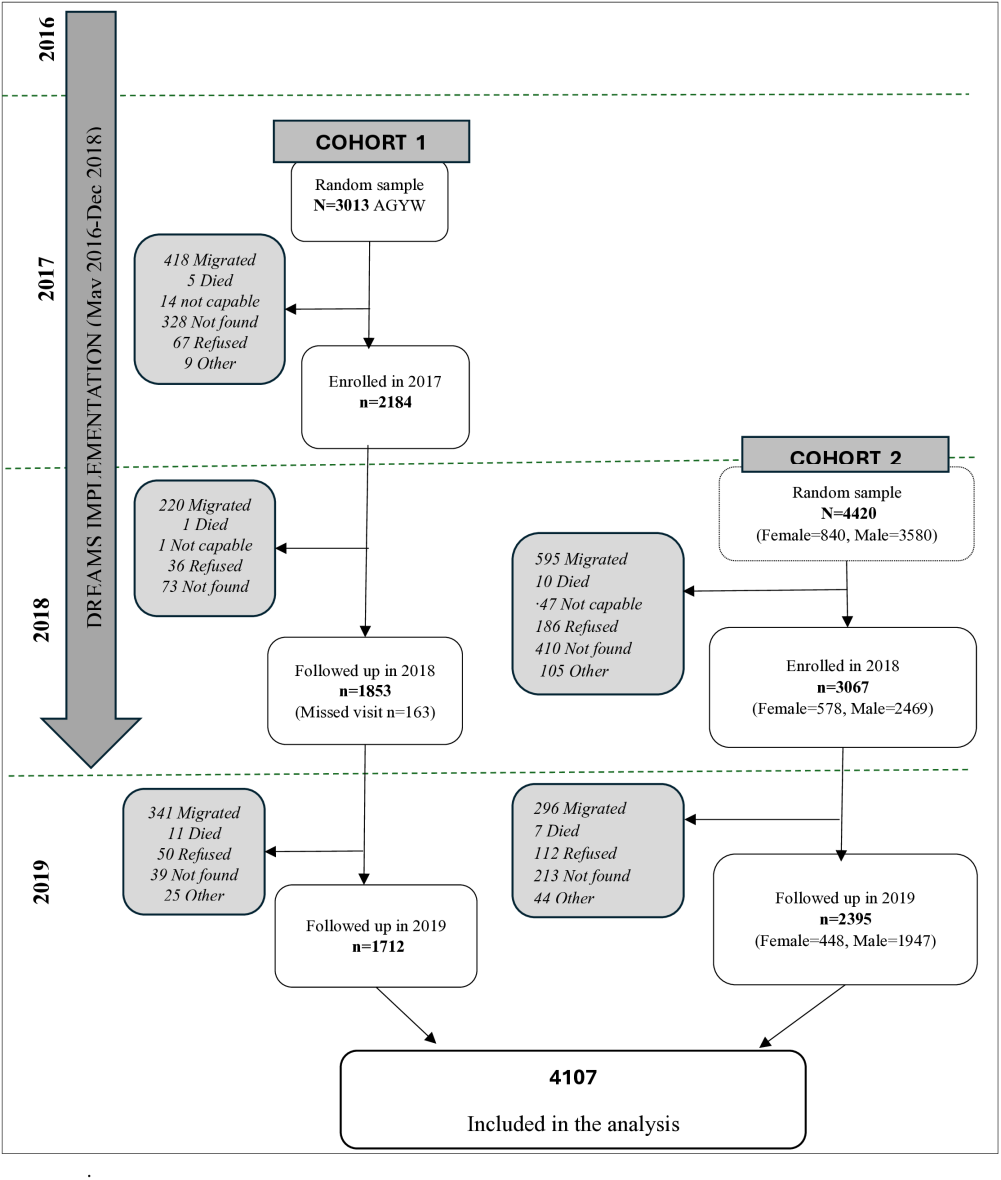
Participants’ recruitment and follow-up.

Of 5251 participants enrolled in both cohorts, 4107 (78.3%) were followed in 2019 including 163 who had missed the 2018 survey. A total of 3944 participants were included in the analysis. In both cohorts, the most common reason for loss to follow-up was migration, as indicated in Fig 2.

The characteristics of participants are described in Table 2. More than half of participants were adolescents aged 13–19 years. About 70% had some secondary education. About a quarter reported food insecurity. Approximately 38% reported experiences of violence, about half reported ever having had sex or ever been pregnant, and 12% tested positive for HIV. The intervention groups differed significantly by several characteristics except for experienced violence. About 20% of participants in the individual-level interventions group reported CMD symptoms above the cut off score of 9.

**Table 2:** Baseline characteristics of participants at baseline.

	All sample (N = 3944)	No intervention (N = 519)	Community-level (N = 1060)	Individual-level (N = 814)	Multi-level (N = 1551)	Chi-square p-value
	n (Column %)	n (Column %)	n (Column %)	n (Column %)	n (Column %)	
**Sex**						<0.001
Female	1997(52.6)	150 (28.9)	536 (50.6)	453 (55.7)	858 (55.3)	
**Age group**						<0.001
13-19	2321 (58.8)	134 (25.8)	973 (91.8)	98 (12.0)	1116 (72.0)	
20-24	719 (18.2)	114 (22.0)	68 (6.4)	270 (33.2)	267 (17.2)	
25-29	641 (16.3)	164 (31.6)	15 (1.4)	328 (40.3)	134 (8.6)	
30-35	263 (6.7)	107 (20.6)	4 (0.4)	118 (14.5)	34 (2.2)	
**Level of education**						<0.001
None or some primary	345 (8.8)	58 (11.3)	132 (12.5)	45 (5.6)	110 (7.1)	
Some secondary	2744 (69.8)	259 (50.3)	907 (85.6)	328 (40.5)	1250 (80.7)	
Completed secondary	843 (21.4)	198 (38.4)	20 (1.9)	436 (53.9)	189 (12.2)	
**Urbanicity**						<0.001
Peri-urban/urban vs Rural	1343 (34.1)	195 (37.6)	325 (30.7)	322 (39.6)	501 (32.3)	
**Household wealth index**						0.001
Low	979 (24.8)	121 (23.3)	265 (25.0)	195 (24.0)	398 (25.7)	
Middle	1323 (33.5)	158 (30.4)	339 (32.0)	258 (31.7)	568 (36.6)	
High	1267 (32.1)	171 (32.9)	368 (34.7)	275 (33.8)	453 (29.2)	
Unknown	375 (9.5)	69 (13.3)	88 (8.3)	86 (10.6)	132 (8.5)	
**Food insecurity**						<0.001
Yes	1015 (25.7)	133 (25.7)	222 (20.9)	223 (27.4)	437 (28.2)	
**CMD**						<0.001
Yes	626 (15.9)	73 (14.1)	112 (10.6)	168 (20.6)	273 (17.6)	
**Ever experienced violence**						0.175
Yes	1494 (37.9)	190 (36.6)	401 (37.8)	287 (35.3)	616 (39.7)	
**Ever migrated**						<0.001
Never	2904 (73.6)	281 (54.1)	994 (93.8)	380 (46.7)	1249 (80.5)	
Within HDSS	350 (8.9)	60 (11.6)	42 (4.0)	100 (12.3)	148 (9.5)	
External migration	690 (17.5)	178 (34.3)	24 (2.3)	334 (41.0)	154 (9.9)	
**HIV status**						<0.001
Negative	3018 (76.5)	290 (55.9)	920 (86.8)	534 (65.6)	1274 (82.1)	
Positive	479 (12.1)	123 (23.7)	50 (4.7)	153 (18.8)	153 (9.9)	
Unknown	447 (11.3)	106 (20.4)	90 (8.5)	127 (15.6)	124 (8.0)	
**Ever had sex, ever pregnant**						<0.001
Never had sex	1910 (48.4)	128 (24.7)	878 (82.8)	67 (8.2)	837 (54.0)	
Ever sex, never pregnant	1168 (29.6)	276 (53.2)	147 (13.9)	375 (46.1)	370 (23.9)	
Ever pregnant	773 (19.6)	101 (19.5)	19 (1.8)	344 (42.3)	309 (19.9)	
Unknown	93 (2.4)	14 (2.7)	16 (1.5)	28 (3.4)	35 (2.3)	

**Fig 3:**
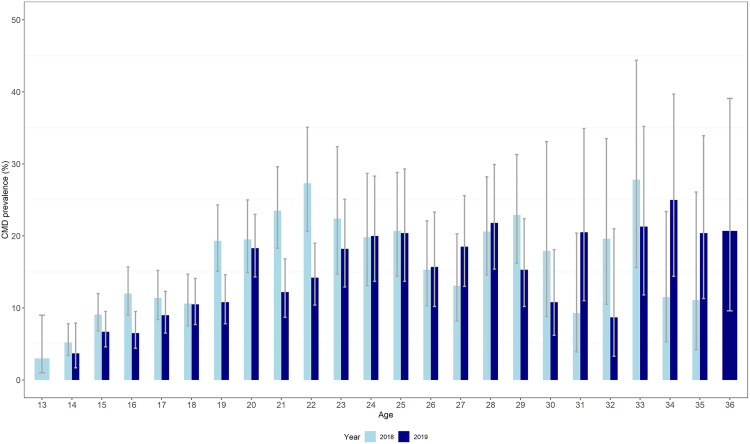
Prevalence estimates (and 95% confidence intervals) of CMD by age and survey year.

The Cronbach alpha for the SSQ-14 was 0.88 suggesting that the responses were consistent between the SSQ-14 items. CMD prevalence increased steadily with age for young people aged below 25 (Fig 3). Overall, there was a decrease in CMD prevalence from 2018 to 2019 among younger participants.

**Fig 4:**
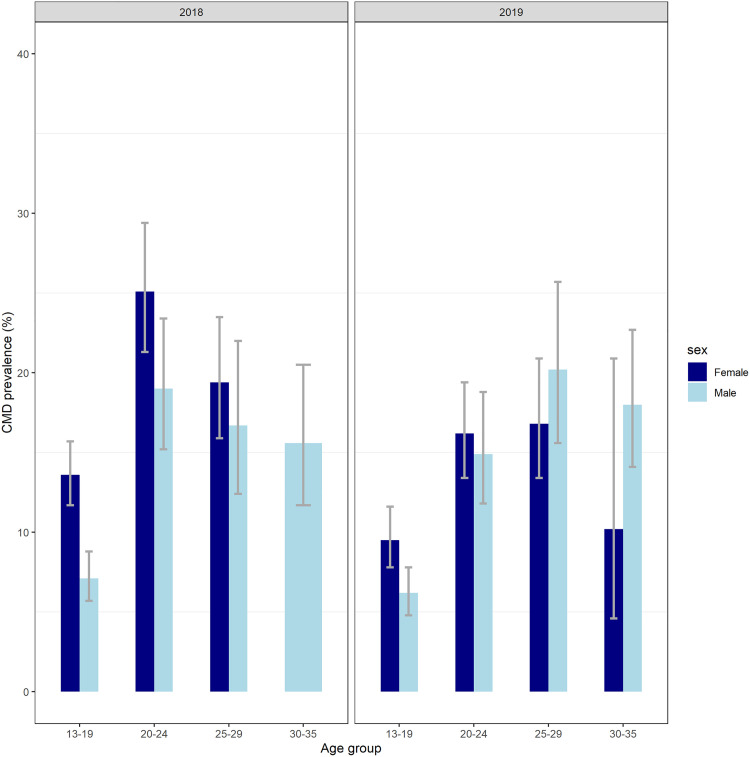
Prevalence estimates (and 95% confidence intervals) of CMD by age, sex and survey year.

In 2018, the prevalence of CMD above the cut off varied by age and sex (Fig 4). It was higher among females compared to males (25.7% vs 18.0 among 20–24-year-olds and 21.3% vs 17.6% among 25–29 year-olds). In 2019, the prevalence was higher among males aged 25–29 (20.2%) and ranged between 6.2-18.0% in other age and sex groups.

In causal analysis, we estimated that participating in individual-level (RD = 7.93, 95%CI: 1.45, 15.0) or multi-level interventions (RD = 4.59, 95% CI: 1.61, 7.78) relative to no intervention significantly increased CMD (Table 3). There was no clear evidence of an effect of community-level interventions alone on CMD (relative to no intervention).

**Table 3:** Estimated average causal effects of multi-level prevention interventions on CMD (Exposure variable with four categories).

	Observed number of young people with CMD (%)	Estimated % of young people with CMD if all received the relevant interventions (95%CI)	Estimated RD (95% CI)
**ALL cohorts**			
No intervention	79 (15.2)	8.70 (6.50, 11.1)	--
Community-level only	84 (7.9)	10.58 (5.33, 17.0)	1.89 (-4.15, 8.62)
Individual-level only	124 (15.2)	16.62 (10.3, 23.3)	7.93 (1.45, 15.0)
Multi-level	197 (12.7)	13.29 (11.3, 15.3)	4.59 (1.61, 7.78)
**AGYW (Cohort 1)**			
No intervention	9 (15.0)	6.56 (2.32, 12.9)	--
Community-level only	41 (7.8)	5.97 (3.96, 8.34)	0.59 (-4.12, 5.59)
Individual-level only	36 (16.9)	16.91 (9.34, 25.4)	10.94 (3.09, 20.0)
Multi-level	101 (13.5)	13.38 (11.0, 16.3)	7.51 (4.23, 11.1)
**ABYM & YW (Cohort 2)**			
No intervention	70 (15.3)	11.26 (8.40, 14.5)	--
Community-level only	43 (8.1)	9.75 (3.41, 21.5)	-1.50 (-8.66, 10.5)
Individual-level only	88 (14.6)	16.62 (9.22, 25.4)	5.37 (-2.31, 14.4)
Multi-level	96 (12.0)	12.84 (10.2, 15.7)	1.59 (-2.67, 5.45)

When comparing multi-level interventions to individual-level interventions alone and community-level interventions alone, there was no significant effect on CMD in the overall sample (Table 4). Among AGYW aged 13–22 (Cohort 1), participating in multi-level interventions relative to community-level interventions was estimated to significantly increase CMD (RD = 5.45, 95% CI: 1.54, 8.92).

**Table 4:** Estimated effect of multi-level interventions on CMD (binary exposure variables comparing multi-level interventions to single-level interventions).

	Observed N with CMD among community/ individual level intervention groups (%) (A)	Estimated % of young people with CMD if all received only individual/community level interventions (95% CI) (B)	Observed N with CMD among multi-level intervention group (%) (C)	Estimated % of young people with CMD if all received multi-level interventions (95% CI) (D)	Estimated Risk difference (95% CI)^a^ (E)
**ALL cohorts**					
Community-level only	84 (7.9)	8.71 (6.25, 12.2)	197 (12.7)	11.5 (9.85, 13.2)	2.79 (-1.07, 5.72)
Individual-level only	124 (15.2)	14.18 (11.88, 16.54)	197 (12.7)	17.4 (12.8, 22.3)	-3.20 (-8.57, 1.92)
**AGYW (Cohort 1)**					
Community-level only	41 (7.8)	7.29 (4.75, 10.4)	101 (13.5)	12.8 (10.5, 15.3)	5.45 (1.54, 8.92)
Individual-level only	36 (16.9)	16.9 (9.99, 24.9)	101 (13.5)	14.4 (11.6, 17.5)	-2.43 (-11.1, 5.09)
**ABYM & YW (Cohort 2)**					
Community-level only	43 (8.1)	9.57 (5.86, 14.9)	96 (12.0)	10.7 (8.72, 12.8)	1.14 (-4.30, 5.29)
Individual-level only	88 (14.6)	16.0 (9.93, 23.4)	96 (12.0)	13.9 (10.9, 16.9)	-2.13 (-9.85, 4.83)

^a^ The estimates in column E are derived by subtracting column B from column D values.

## Discussion

In this study, we estimated the prevalence of CMD above the cut off score of 9, and the effect of HIV prevention interventions on common mental disorders among adolescents and young adults in rural South Africa. The prevalence of CMD was high, especially among females compared to males, and increased with age. The multivariable analysis showed that, it was associated with older age, and screening positive for CMD in the previous surveys. Exposure to individual-level interventions only or multi-level interventions compared to no intervention was found to increase CMD in young people.

The high prevalence of CMD and its association with age have been reported in the previous study conducted in this setting [34]. High levels of CMD in young adults may be the result of undiagnosed or untreated CMD during adolescence. As adolescents transition into adulthood, they are exposed to several social stressors including lack of economic opportunities. The situation may have been worsened by the lack of interventions specific to mental health (e.g., counselling), or those that address some of the precursors or drivers of mental health challenges in the first place. A systematic review that looked at the effect of multi-level interventions on CMD among AYA in sub-Saharan Africa found that interventions that were targeting vulnerable adolescents such as participants in this study included individual-level interventions tailored to individual needs [35]. Participants in this study would have benefited from such interventions as some of them were already affected by mental health problems.

In this study, we found no evidence that multi-level interventions reduce CMD among adolescents and young adults. The most likely explanation is that the interventions under study were not specifically designed to address mental health problems. While some components of DREAMS such as social asset building and social protection may have had indirect benefits on participants’ overall well-being, the program did not include targeted mental health interventions such as counselling, psychological therapy, or structured psychosocial support. As such, the lack of impact on mental health outcomes among participants may reflect the fact that mental health was not a primary focus of the program design or implementation. Future programming aiming to improve mental health outcomes in similar populations should incorporate dedicated, evidence-based mental health components to achieve measurable effects.

The finding that individual-level interventions (either alone or combined with community-level interventions) increase CMD compared to no intervention is contrary to a previous study that found depression to be associated with non-utilisation of HIV testing and counselling services [36]. In this setting, it could mean that participants who received multi-level interventions or healthcare interventions only were more vulnerable and might have had other health issues that were not accounted for in the analysis, compared to other groups. Furthermore, we cannot speculate that HIV status resulting from participation in individual-level interventions in this study is a cause for CMD, as evidence from previous research indicates that depression and distress are common among individuals seeking HIV testing, even before receiving their HIV test results [37]. As HIV testing and counselling services were primarily targeted at vulnerable populations, there may be other health conditions or health needs that could not be addressed by these interventions [29]. Thus, mental health support should be provided not only for young people already known to be living with HIV but also for those seeking HIV testing and counselling services.

While community-level interventions such as social protection (cash transfers and parenting) and social assets are designed to empower young people to be able to deal with social challenges, in this study they did not influence mental health when compared to no intervention in the overall sample. The possible explanation could be that these interventions may require a higher dose or longer duration to be able to achieve their effect on mental health outcomes; and that vulnerable youth may need extra social support not afforded by these interventions to cope with their challenges including preexisting CMD. Furthermore, since the social assets building interventions were primarily targeted at adolescent girls and young women, combining this group with older women and adolescent boys and young men in the same analysis may have diluted the observed effect of the community-level interventions. Additionally, the discontinuation of DREAMS in mid-2018 could have affected the effectiveness of interventions (especially those that were newly introduced through DREAMS, e.g., violence prevention, empowerment, parenting and social assets) on the outcomes measured afterward. This suggests that HIV prevention interventions without the appropriate mental health interventions such as cognitive behavioural therapy may not be useful in addressing mental health issues among young people in this setting.

In contrast to the overall findings, community-level interventions alone were found to be protective against CMD among AGYW when compared with multi-level interventions. The possible explanation could be that AGYW who received multi-level interventions were more vulnerable, as DREAMS interventions particularly the health-care interventions (e.g., HIV testing and post-violence care) targeted high-risk AGYW, potentially attenuating the observed protective effect compared to those who received only community-level interventions. Moreover, community-level interventions may have had broader reach for this group, and some of AGYW may have benefited from social assets building interventions specifically designed to build resilience among them. Evidence from Gourlay et al. who evaluated the DREAMS program, supports this mechanism, showing that AGYW who participated in DREAMS had increased social support and self-efficacy which are key protective factors for mental health and care engagement [18].

We acknowledge that culturally and contextually specific factors within the rural South African setting may have influenced both the prevalence of CMD and effectiveness of interventions. Persistent societal stigma surrounding mental health and HIV often discourages disclosure and help-seeking, thereby limiting access to mental health support, especially in rural areas where the burden of misconceptions and spiritual interpretations further inhibits care-seeking behaviour [38,39]. In these settings, limited access to mental health services, long travel distances, and shortages of trained professionals further constrain uptake and continuity of care [40]. Additionally, social norms surrounding emotional expression and gendered expectations may have influenced how CMD symptoms are experienced, understood and reported by males [41,42].

In light of these contextual influences on mental health, culturally grounded, community-based mental health interventions have shown promising outcomes for adolescents living with HIV in sub-Saharan Africa. In South Africa, the VUKA family-based program which integrates HIV education with coping and communication skills has been associated with improved mental health outcomes and reduced risk behaviours [43]. The Amagugu intervention, supporting HIV positive mothers in disclosing their status to their children has demonstrated positive effects on children’s emotional well-being and family communication [44]. These examples highlight the potential of community-based approaches to promote mental health in contexts with limited access to formal services. They also suggest that, when culturally adapted and embedded within communities, such interventions may complement or even offer an alternative to more complex multi-level models.

## Limitations

This analysis has some limitations. First, as already stated, the interventions were not randomly assigned to groups, and our results may have been influenced by unmeasured confounding. Important factors such as prior trauma exposure or related health conditions were not available in our dataset, yet these could have influenced both the likelihood of adolescents receiving specific interventions and their risk of developing common mental disorders (CMD). For instance, adolescents with higher psychosocial vulnerability might have been more likely to receive individual-level interventions and more prone to CMD, potentially biasing our results away from the null. This is supported by the sensitivity analysis (S2 Table and S3 Table) where the observed associations for individual-level and multi-level interventions had relatively low E-values (less than 2), suggesting that even modest unmeasured confounding could explain these findings. This limitation highlights the constraints of the causal inference methods used in this study, which cannot fully adjust for unobserved factors.

Second, grouping certain interventions (e.g., social protection and social assets) may have diluted or masked the effects of specific components, biasing estimates toward the null. Third, we used a self-reported data, which may be subject to recall bias and social desirability, potentially leading to underreporting of stigmatised conditions and underestimation of CMD prevalence. Fourth, loss to follow-up was notably higher among participants aged 20–24, those who had completed secondary school, had ever out-migrated and were sexually active (S4 Table). This differential loss to follow-up may have introduced selection bias and overestimation of outcomes. However, given our large sample size and overall retention rate, the impact of this bias on the study findings is likely limited. Furthermore, although residual confounding and other limitations inherent to observational data remain, the consistency in the results across sensitivity analyses strengthens the robustness of our findings. Fifth, these interventions were delivered by implementing partners and so we cannot comment on the fidelity of some of the curriculum-based interventions and inconsistent implementation may have reduced the effectiveness of interventions. Lastly, the SSQ-14 has not specifically been validated for use in adolescents under 18 years which may have led to misclassification and either under- or overestimation of CMD prevalence in this group.

## Conclusions

In this analysis, we have identified groups of young people with common mental disorders and showed that multi-level HIV prevention interventions without relevant mental health components are insufficient to meet their care needs. These findings suggest that mental health screening should be prioritised for young people accessing HIV-related services to ensure early diagnosis and treatment of CMD. Integrating brief, validated mental health screening tools [e.g., adapted versions of the SSQ or Patient Health Questionnaire (PHQ-9)] into routine HIV prevention and treatment services could facilitate early identification of adolescents at risk of CMD. Interventions should also consider culturally adapted counselling approaches such as task-shifting models that train lay counsellors or peer mentors to deliver structured, low-intensity psychosocial support grounded in local idioms of distress. Additionally, incorporating mental health components into existing HIV prevention packages such as adherence clubs, family strengthening programs, or youth-friendly clinic services, could improve both mental health and HIV-related outcomes. These context-specific strategies can enhance feasibility, scalability, and relevance for policymakers and implementers working to support adolescent well-being in similar low-resource settings.

Since this study used observational data, there may be unmeasured confounders that were not controlled for; therefore, dismantling studies that test individual components like cash transfers or psychosocial support can reveal which are most effective for improving mental health. This can guide the development of simpler, cost-effective interventions that still deliver strong results. Future research should explore how interventions improve mental health in young people, focusing on protective factors like social support, caregiver communication and stigma reduction.

## Supporting information

S1 Fig Distribution of propensity scores for overall sample. (TIF)

S2 Fig Distribution of propensity scores for Cohort 1. (TIF)

S3 Fig Distribution of propensity scores for Cohort 2. (TIF)

S1 Table Covariate balance diagnostics for overall sample. (DOCX)

S4 Fig Covariate balance diagnostics for overall sample. (TIF)

S5 Fig Covariate balance diagnostics for Cohort 1. (TIF)

S6 Fig Covariate balance diagnostics for Cohort 2. (TIF)

S2 Table Sensitivity analysis (E-values) for main analysis. (DOCX)

S3 Table Sensitivity analysis (E-values) for sub-analysis. (DOCX)

S4 Table Factors associated with loss to follow-up. (DOCX)
