# Supplementary material for: The effect of multi-level HIV prevention interventions on common mental disorders among adolescents and young adults in rural South Africa
Source: PLOS Glob Public Health. 2025 Dec 11;5(12):e0005183. doi: 10.1371/journal.pgph.0005183 (PMC12697961; doi:10.1371/journal.pgph.0005183)
Supplement: S1 Table — (DOCX) [file pgph.0005183.s004.docx]

S1 Table . Covariates balance diagnostic test

|  | **Standardised differences** | | **Variance ratio** | |
| --- | --- | --- | --- | --- |
|  | **Raw** | **Weighted** | **Raw** | **Weighted** |
|  |  |  |  |  |
| **Community-level only** |  |  |  |  |
| Age | -1.6738 | 0.3097 | 0.1224 | 1.4023 |
| Sex | 0.4517 | -0.0690 | 1.2131 | 0.9864 |
| Education | -0.7137 | 0.2030 | 0.3134 | 0.6581 |
| Household wealth index | -0.1051 | -0.0463 | 0.8907 | 1.0864 |
| Urbanicity | -0.1431 | -0.0934 | 0.9064 | 0.9292 |
| Food insecurity | -0.1146 | -0.0187 | 0.8648 | 0.9776 |
| Baseline CMD | -0.1036 | -0.0170 | 0.7863 | 0.9677 |
| Violence | 0.0329 | -0.2081 | 1.0166 | 0.8626 |
| Migration | -1.0309 | 0.1459 | 0.1452 | 1.2443 |
| Sexual behaviour | -0.7795 | 0.4097 | 0.6200 | 3.9552 |
| HIV status | -0.3512 | 0.0034 | 0.4752 | 1.0181 |
|  |  |  |  |  |
| **Individual-level only** |  |  |  |  |
| Age | 0.1066 | 0.1891 | 0.6612 | 0.9771 |
| Sex | 0.5544 | 0.1573 | 1.1995 | 0.9951 |
| Education | 0.3377 | 0.1342 | 0.8533 | 1.0659 |
| Household wealth index | -0.0538 | 0.0229 | 0.9336 | 1.1060 |
| Urbanicity | 0.0447 | 0.0121 | 1.0208 | 1.0083 |
| Food insecurity | 0.0341 | 0.1405 | 1.0361 | 1.1498 |
| Baseline CMD | 0.1741 | 0.0746 | 1.3554 | 1.1398 |
| Violence | -0.0222 | -0.1198 | 0.9863 | 0.9296 |
| Migration | 0.1551 | 0.1673 | 1.0354 | 1.1825 |
| Sexual behaviour | 0.3627 | 0.2915 | 1.0298 | 0.8547 |
| HIV status | -0.1189 | 0.0507 | 0.8223 | 1.1176 |
|  |  |  |  |  |
| **Multi-level** |  |  |  |  |
| Age | -1.1399 | 0.0959 | 0.4590 | 1.2519 |
| Sex | 0.5526 | 0.0348 | 1.1992 | 1.0031 |
| Education | -0.3956 | 0.1966 | 0.4505 | 0.6949 |
| Household wealth index | -0.1639 | 0.0531 | 0.8748 | 1.0250 |
| Urbanicity | -0.1108 | 0.0511 | 0.9299 | 1.0322 |
| Food insecurity | 0.0509 | 0.0633 | 1.0529 | 1.0713 |
| Baseline CMD | 0.0967 | 0.0267 | 1.1986 | 1.0502 |
| Violence | 0.0700 | 0.0468 | 1.0337 | 1.0204 |
| Migration | -0.6421 | 0.1074 | 0.4797 | 1.1137 |
| Sexual behaviour | -0.2682 | 0.0362 | 1.0549 | 1.0398 |
| HIV status | -0.3678 | -0.0012 | 0.4482 | 1.0030 |
