# Supplementary material for: The effect of multi-level HIV prevention interventions on common mental disorders among adolescents and young adults in rural South Africa
Source: PLOS Glob Public Health. 2025 Dec 11;5(12):e0005183. doi: 10.1371/journal.pgph.0005183 (PMC12697961; doi:10.1371/journal.pgph.0005183)
Supplement: S2 Table — (DOCX) [file pgph.0005183.s008.docx]

S2 Table. Effect estimates and E-values (Multi-level vs no intervention)

|  | **IPTW Estimated RD (95% CI)** | **PS-regression adjustment Estimated RD (95% CI)** | **E-value** |
| --- | --- | --- | --- |
| **ALL cohorts** |  |  |  |
| No intervention | -- |  |  |
| Community-level only | 1.89 (-4.15, 8.62) | -1.54 (-6.57, 3.81) | 2.43 |
| Individual-level only | 7.93 (1.45, 15.0) | 4.44 (-2.69, 12.8) | 1.03 |
| Multi-level | 4.59 (1.61, 7.78) | 2.38(-1.34, 6.20) | 1.69 |
| **AGYW (Cohort 1)** |  |  |  |
| No intervention | -- |  |  |
| Community-level only | 0.59 (-4.12, 5.59) | 0.66 (-15.2, 14.8) | 3.27 |
| Individual-level only | 10.94 (3.09, 20.0) | 5.95 (-12.3, 23.0) | 1.5 |
| Multi-level | 7.51 (4.23, 11.1) | 7.08 (-8.74, 20.3) | 1.47 |
| **ABYM & YW (Cohort 2)** |  |  |  |
| No intervention | -- |  |  |
| Community-level only | -1.50 (-8.66, 10.5) | -0.47 (-8.68, 11.2) | 3.19 |
| Individual-level only | 5.37 (-2.31, 14.4) | 5.29 (-3.75, 15.6) | 1.25 |
| Multi-level | 1.59 (-2.67, 5.45) | 2.23 (-1.89, 6.07) | 1.86 |
