# Supplementary material for: The effect of multi-level HIV prevention interventions on common mental disorders among adolescents and young adults in rural South Africa
Source: PLOS Glob Public Health. 2025 Dec 11;5(12):e0005183. doi: 10.1371/journal.pgph.0005183 (PMC12697961; doi:10.1371/journal.pgph.0005183)
Supplement: S3 Table — (DOCX) [file pgph.0005183.s009.docx]

S3 Table. Effect estimates and E-values (Multi-level vs community/individual-level)

|  | **IPTW Estimated RD (95% CI)** | **PS-regression adjustment Estimated RD (95% CI)** | **E-value** |
| --- | --- | --- | --- |
| **ALL cohorts** |  |  |  |
| Community-level only | 2.79 (-1.07, 5.72) | 2.03 (-0.48, 4.27) | 2.59 |
| Individual-level only | 3.20 (-8.57, 1.92) | 0.06 (-4.16, 4.13) | 1.64 |
| **AGYW (Cohort 1)** |  |  |  |
| Community-level only | 5.45 (1.54, 8.92) | 4.24 91.00, 7.65) | 2.86 |
| Individual-level only | -2.43 ( -11.1, 5.09) | -1.27 (-8.40, 5.19) | 1.82 |
| **ABYM & YW (Cohort 2)** |  |  |  |
| Community-level only | 1.14 (-4.30, 5.29) | 0.93 (-2.38, 3.94) | 2.33 |
| Individual-level only | -2.13 (-9.85, 4.83) | 1.05 (-4.10, 6.20) | 1.75 |
